# Liraglutide Potently Protects Against Streptozotocin-Induced Islet Injury Associated with Inhibition of HMGB1 Release

**DOI:** 10.3390/cells15131203

**Published:** 2026-07-02

**Authors:** Yuzhen Shi, Xi Yang, Xiaoping Luo, Jun Yang, Yong Zhang, Gang Chen, Ling Hou

**Affiliations:** 1 Department of Pediatrics, Tongji Hospital, Tongji Medical College, Huazhong University of Science and Technology, Wuhan 430030, China; 2 Hubei Key Laboratory of Pediatric Genetic Metabolic and Endocrine Rare Diseases, Wuhan 430030, China; 3 Hubei Provincial Clinical Research Center for Child Growth, Development, and Metabolic Diseases, Wuhan 430030, China; 4 Institution of Organ Transplantation, Tongji Hospital, Tongji Medical College, Huazhong University of Science and Technology, Wuhan 430030, China; 5 Key Laboratory of Organ Transplantation, Ministry of Education, Ministry of Public Health, Chinese Academy of Medical Sciences, Wuhan 430030, China

**Keywords:** streptozotocin, glucagon-like peptide-1 receptor agonist, liraglutide, islet beta cells, high mobility group protein B1

## Abstract

**Highlights:**

**What are the main findings?**

1. Liraglutide potently reduces STZ-induced islet injury by inhibiting HMGB1 release via GLP-1 receptor activation.
2. Liraglutide suppresses pancreatic TLR4 expression and reduces pro-inflammatory cytokines (IFN-γ, IL-1β, CXCL10) in vivo.

**What are the implications of the main findings?**

1. This study identifies HMGB1 as a key downstream target of GLP-1 receptor signaling in the protection against islet injury.
2. Liraglutide may serve as a potential protective agent for preventing islet damage in conditions such as type 1 diabetes or islet transplantation.

**Abstract:**

It is unknown whether the glucagon-like peptide-1 (GLP-1) receptor agonists have a significant protective effect against acute islet injury. High mobility group box 1 (HMGB1) is a damage-associated molecular pattern (DAMP) molecule released from stressed or injured pancreatic β-cells, which triggers inflammatory responses through toll-like receptor 4 (TLR4) signaling. This study investigated the protective effect and mechanism of liraglutide on acute islet injury induced by low doses of streptozotocin (STZ). The results showed that liraglutide pretreatment preserved the structural integrity of pancreatic islets, improved insulin levels and glucose tolerance, and significantly reduced the incidence of diabetes in STZ-treated mice. Liraglutide was also found to inhibit STZ-induced release of HMGB1 and reduce the expression of TLR4 and inflammatory factors IFN-γ, IL-1β, and CXCL10. Moreover, administration of exogenous HMGB1 or antagonism of the GLP-1 receptor diminished liraglutide’s protective effects. These findings suggest that liraglutide has a strong protective effect on STZ-induced acute islet injury, most likely through the inhibition of HMGB1 release, which provides an experimental basis for the application of liraglutide as a protective agent for acute islet injury.

## 1. Introduction

Liraglutide is a GLP-1 receptor agonist (GLP-1RA) mainly used in the treatment of type 2 diabetes mellitus. In addition to enhancing pancreatic β-cell function by increasing insulin synthesis and secretion, liraglutide has been shown to increase the mass of pancreatic β-cells by promoting β-cell proliferation and regeneration and decreasing apoptosis through multiple signaling pathways [1,2]. Several studies have reported that liraglutide attenuates apoptosis induced by reactive oxygen species, cytokines, high glucose, and high fat in β-cell lines or pancreatic β-cells of diabetic mice [3,4,5]. Its mechanisms of action may involve alleviating endoplasmic reticulum stress, regulating autophagy levels, maintaining oxidative stress homeostasis, and inhibiting inflammation [6]. For example, Shimoda M et al. reported that treatment of type 2 diabetic db/db mice with liraglutide inhibited glucolipotoxicity-induced islet oxidative stress and endoplasmic reticulum stress, and attenuated islet cell apoptosis [7]. Nanjing Guo et al. found that liraglutide upregulated anti-apoptotic protein Bcl-2 levels and downregulated pro-apoptotic protein Bax levels in the islet cells of rats, delaying the onset of chronic spontaneous type 2 diabetes [8]. While liraglutide has been shown to be protective against chronic damage to islets in these models, whether it is protective against acute damage to islets is unclear.

Streptozotocin (STZ) is a chemical drug that selectively destroys pancreatic islet β-cells, primarily by entering β-cells mediated by glucose transporters and inducing DNA alkylation damage, excessive ROS production, and lipid peroxidation [9]. Several prior studies demonstrate that exposure to STZ results in glutathione depletion, severe oxidative stress, and acute cell death in pancreatic islet β-cells [10,11]. Since liraglutide has shown a strong protective effect on acute kidney and intestinal injuries caused by ischemia–reperfusion [12,13], it may also have a significant protective effect against STZ-induced acute islet cell injury.

High mobility group protein B1 (HMGB1) is a nuclear protein that regulates gene transcription and participates in DNA repair [14]. It has been found to be strongly correlated with pancreatic islet β-cell damage [15]. HMGB1 is released into the extracellular space as a “danger signal” in response to damage or stress, acting as a pro-inflammatory factor that can trigger the aggregation of inflammatory cells through chemotaxis and induce apoptosis of pancreatic islets. The amount of HMGB1 released reflects the extent of islet damage [16]. In addition, several studies have reported that GLP-1 receptor agonists can improve or protect from the disease by affecting the expression and distribution of HMGB1 [17,18]. GLP-1 receptor agonists ameliorate pancreatic β-cell apoptosis in T2DM, improve islet function, and suppress islet transplant rejection, all of which are mediated by the inhibition of islet inflammation [19,20,21]. Therefore, this study investigated whether liraglutide exerts a protective effect against STZ-induced pancreatic islet injury that is associated with inhibition of HMGB1 release.

## 2. Methods

### 2.1. Animals

Six-week-old male C57BL/6J (18–22 g) mice were purchased from Beijing Vitalriver Co., Ltd. (Beijing, China). They had free access to water and food for 12 h light–dark cycle and were maintained in specific pathogen-free conditions (Temperature: 20–24 °C). Experiments were approved by the Institutional Animal Care and Use Committee of Tongji Medical College, Huazhong University of Science and Technology (protocol code TJH-202110016 and date of approval 16 October 2021).

Randomization and blinding: After one week of acclimation feeding, the mice were randomly assigned to different groups using a random number table. Group allocation was performed by an investigator not involved in the subsequent experiments and sealed in opaque envelopes (allocation concealment). This study employed a double-blind design. Drug preparation and administration were carried out by different personnel, and the individual performing the injections was only aware of the syringe codes. And data analysis were all conducted by investigators blinded to the group allocation.

### 2.2. Cell Culture

NIT-1 cells (a mouse insulinoma cell line) were purchased from Bena Culture Co., Ltd. (Beijing, China) and cultured in 1640 medium (Gibco, Grand Island, NY, USA) containing 10% fetal bovine serum (Gibco, Grand Island, NY, USA) with 1% antibiotics (100 U/mL penicillin and 100 μg/mL streptomycin).

### 2.3. Drug Administration

**Figure 1:**
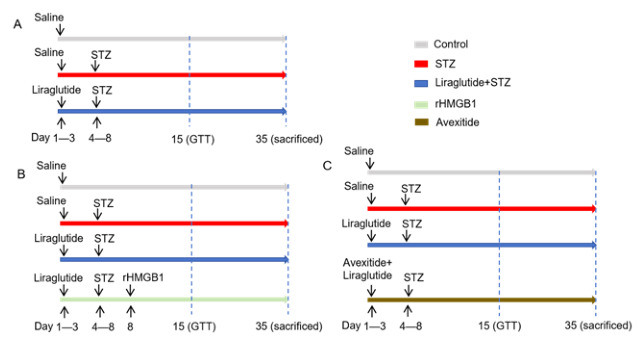
The schedule of the animal experiment for the different treatments. (**A**) Experimental timeline for liraglutide pretreatment and STZ-induced diabetes modeling. (**B**) Experimental timeline for rHMGB1 administration. (**C**) Experimental timeline for avexitide administration.

Following a one-week acclimation period, the mice were randomly divided into three groups: a control group, STZ group, and Liraglutide + STZ group. On days 1–3, the Liraglutide + STZ group received subcutaneous injections of liraglutide (Novo Nordisk, Bagsværd, Denmark) at a dose of 250 μg/kg at 12 h intervals, while the control and STZ groups received saline injections [22,23]. On days 4–8, the STZ and Liraglutide + STZ groups were injected with STZ (S0130, Sigma-Aldrich, St. Louis, MO, USA) at a dose of 45 mg/kg at 24 h intervals, while the control group received citrate buffer injections. Mice with 12 h fasting blood glucose levels > 11.1 mmol/L were regarded as diabetic [24]. The schedule of the animal experiment for the different treatments above is shown in Figure 1A.

To investigate the role of HMGB1, the mice were randomly divided into four groups: a control group, STZ group, Liraglutide + STZ group and rHMGB1 group. In the rHMGB1 group, rHMGB1 (1690-HMB-050, R&D Systems, Minneapolis, MN, USA) was injected via the tail vein at a dose of 20 μg per mouse on day 8, after the Liraglutide and STZ injection was completed [25]. The schedule of the animal experiment for the different treatments above is shown in Figure 1B.

In addition, the mice were randomly divided into four groups: a control group, STZ group, Liraglutide + STZ group and Avexitide group. In the Avexitide group, avexitide (HY-P0264, MCE, Monmouth Junction, NJ, USA) was injected subcutaneously at a dose of 250 μg/kg 30 min before each liraglutide administration [12]. The schedule of the animal experiment for the different treatments above is shown in Figure 1C.

### 2.4. Western Blot

A Cytoplasmic and Nuclear Protein Kit (P0028, Biyun Tian, Shanghai, China) was used to extract cytoplasmic and nuclear proteins from pancreatic or NIT-1 cells. Protein concentrations were measured using a BCA Protein Assay Kit (P0012, Biyun Tian, China). Proteins were separated on a 10% SDS-PAGE gel and blotted onto a PVDF membrane (Bio-Rad Inc., Hercules, CA, USA) that was blocked in bovine serum albumin (5%). The membranes were incubated with rabbit anti-HMGB1 monoclonal primary antibody (ab79823, Abcam, Cambridge, MA, USA) overnight at 4 °C, washed, and then incubated with horseradish peroxidase-coupled secondary antibody (GB23303, Servicebio, Wuhan, China) for 1 h at room temperature. The expression levels of target proteins were normalized to β-actin (66009-1-Ig, Proteintech, Wuhan, China) or Histone-H3 (17168-1-AP, Proteintech, China). Proteins were visualized by enhanced chemiluminescence (BMU102, Abbkine, Wuhan, China).

### 2.5. Enzyme-Linked Immunosorbent Assay (ELISA)

Mice were subjected to retro-orbital blood collection under anesthesia. Blood samples were allowed to clot at room temperature for 30 min, then centrifuged at 8000 rpm for 15 min in a centrifuge pre-cooled to 4 °C. The supernatant serum was then collected and stored at −80 °C, which will be used for an Enzyme-Linked Immunosorbent Assay (ELISA). ELISA kits were used to detect HMGB1 (F10620, Xitang, Changsha, China), insulin (CSB-E05071m, CUSABIO, Wuhan, China), IL-1β (RK00006, ABclonal, Wuhan, China), IFN-γ (RK00019, ABclonal, China), and CXCL10 (RK00056, ABclonal, Wuhan, China). All serum samples were diluted according to the dilution ratios recommended by the manufacturer. As stated in the kit manufacturer’s manual, the assay exhibited good detection sensitivity with a coefficient of variation (CV) below 10%. Each sample was assayed in triplicate, and the final result was taken as the average of the three replicates. The standard provided with the kit was used to construct a four-parameter logistic (4-PL) standard curve by plotting standard concentrations against their corresponding absorbance values, with an R^2^ > 0.99 considered acceptable.

### 2.6. Histopathologic and Immunohistochemistry (IHC) Staining

The mouse pancreas was fixed in 4% paraformaldehyde for 24–48 h. After paraffin embedding, the pancreatic tissue was sliced into 4 μm thick sections. The sections were stained with eosin and hematoxylin (HE) to visualize the islet structure. Insulin antibodies (GB13121-50, Servicebio, China), HMGB1 (ab79823, Abcam, USA), TLR2 (17236-1-AP, Proteintech, China), AGER (16346-1-AP, Proteintech, China), and TLR4 (66350-1-Ig, Proteintech, China) were used for immunohistochemistry.

### 2.7. Immunofluorescence Staining

Cell slides were fixed with 4% paraformaldehyde, permeabilized with 0.5% Triton X-100, and enclosed in 5% bovine serum albumin. They were then incubated with primary antibody HMGB1 (ab79823, Abcam, USA) overnight at 4 °C. Next, the slides were incubated with FITC-labelled goat anti-rabbit antibody (AS01, ABclonal, China) and Phalloidin (40734ES75, Yeasen, Shanghai, China) for 45 min at room temperature, and DAPI (G1012, Servicebio, China) was used for nuclear staining. Fluorescence microscopy was used to observe and take pictures.

### 2.8. Glucose Tolerance Test (GTT)

The GTT in mice was conducted as previously described [26]. Briefly, mice were fasted for 16 h before the glucose tolerance test with free access to water. On the test day, the mice were weighed and intraperitoneally injected with glucose at a dose of 2 g per kg of body weight. The blood glucose level before glucose injection was measured as the 0 min baseline level. Subsequently, blood glucose levels were measured at 15, 30, 60, 90, and 120 min after glucose injection. Blood glucose was monitored using a glucometer (Roche Diagnostics, Basel, Switzerland).

### 2.9. Real-Time Quantitative Polymerase Chain Reaction

Total RNA was extracted using TRIzol reagent (Takara, Kusatsu, Japan) and reverse-transcribed using a cDNA synthesis reagent kit (Takara, Japan). RT-qPCR was performed using SYBR Green qPCR Master Mix (Vazyme, Nanjing, China). Primer sequences designed for PCR amplifications are listed in Supplementary Table S1.

### 2.10. Statistical Analysis

Data were analyzed using GraphPad Prism software, version 8.0.2 (GraphPad Software, San Diego, CA, USA). Data were expressed as mean ± standard deviation (SD). Two-tailed paired Student’s *t*-test was used to compare the means between two groups. One-way analysis of variance was used for comparisons among multiple groups. Non-parametric tests were applied when the data did not conform to a normal distribution. Homogeneity of variance was evaluated using the Brown–Forsythe test in GraphPad Prism. Given that the assumption of equal variances was met, we performed Tukey’s Honestly Significant Difference (HSD) test for all pairwise comparisons following the significant one-way ANOVA. *p* < 0.05 was taken to indicate statistical significance.

## 3. Results

### 3.1. Characterization of STZ-Induced Islet Injury and Diabetes Model

**Figure 2:**
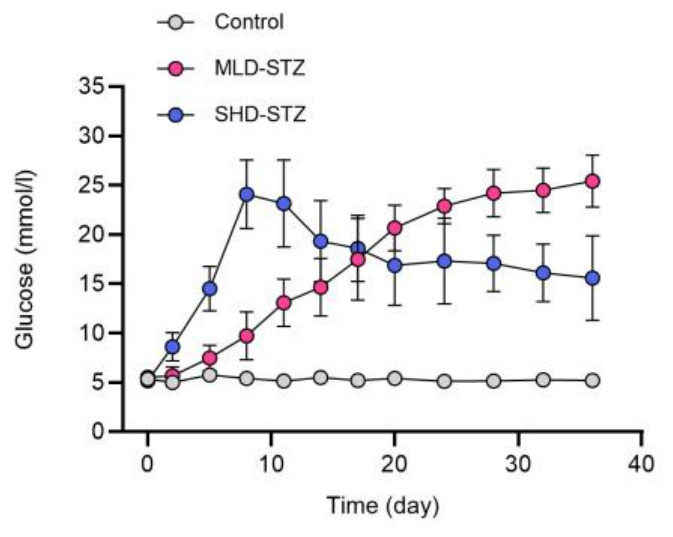
Effects of single high dose (SHD) and multiple low dose (MLD) streptozotocin injections on fasting blood glucose (*n* = 8).

In this study, a pancreatic islet injury model was constructed using STZ in 6-week-old male C57BL/6J mice. It is commonly used to produce animal models of type 1 diabetes mellitus. STZ was administered as a single high dose (one dose, 150 mg/kg) or multiple low doses (five doses, 45 mg/kg). The blood glucose of mice injected with multiple low doses of STZ gradually increased and stabilized. In contrast, the blood glucose of mice injected with a single high dose of STZ increased rapidly; a tendency for blood glucose to fall back was observed at a later stage, resulting in unstable blood glucose levels (Figure 2). Therefore, subsequent experiments used multiple low doses to induce acute islet injury to led to diabetes in mice.

### 3.2. Liraglutide Potently Reduces STZ-Induced Pancreatic Islet Injury and Diabetes

**Figure 3:**
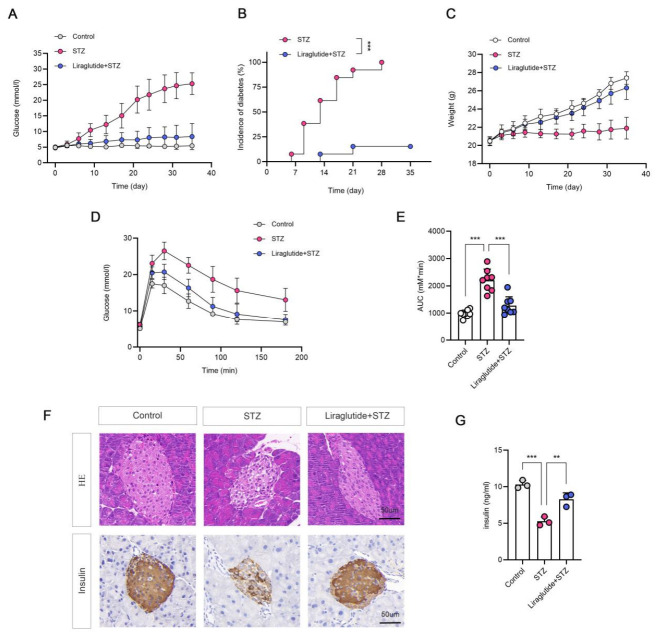
Protective effect of liraglutide on STZ-induced pancreatic islet injury and diabetes. (**A**) Fasting blood glucose levels of mice in control, STZ, and Liraglutide + STZ groups (*n* = 13). (**B**) Incidence of diabetes mellitus in STZ and Liraglutide + STZ groups (*n* = 13). (**C**) Body weight of mice, by group (*n* = 13). (**D**) Glucose tolerance test: blood glucose levels of mice were recorded before and 15, 30, 60, 90, and 120 min after glucose injection (*n* = 8). (**E**) Comparison of area under the curve, by group (*n* = 8). (**F**) Mouse pancreatic tissues were examined by HE staining (bar = 50 μm) and immunohistochemistry (bar = 50 μm) to analyze pancreatic islet structure of and insulin content. (**G**) ELISA was used to detect insulin in each group (*n* = 3). ** *p* < 0.01, *** *p* < 0.001.

The mice were randomly divided into control, STZ, and Liraglutide + STZ groups. Following drug injection, blood glucose showed an overall increasing trend in the STZ group. In contrast, the blood glucose of the Liraglutide + STZ group slightly increased and was similar to the control group (Figure 3A). Twenty-eight days post-injection, all of the mice in the STZ group developed diabetes, whereas only 15.4% (2/13) of the mice in the Liraglutide + STZ group developed diabetes. Furthermore, no other mice developed diabetes in the Liraglutide + STZ group at the end-point of observation, i.e., 35 days post-injection (Figure 3B). Body weight remained similar between the Liraglutide + STZ and control groups, whereas the STZ group exhibited slow weight gain (Figure 3C). Glucose tolerance testing revealed that glucose tolerance was significantly impaired in the STZ group, and the area under the curve was significantly higher than in the other two groups (Figure 3D,E). Analyses of islet structure and insulin content revealed that compared with the control group, the STZ group had decreased islet cell volume, disordered internal structure, and significantly reduced insulin positive staining and serum insulin levels. In contrast, the Liraglutide + STZ group’s islet structure was more intact, with a larger number of internal cells, and relatively abundant cytoplasm (Figure 3F). Furthermore, serum insulin content was higher than in the STZ group (Figure 3G).

### 3.3. Liraglutide Inhibits STZ-Induced Pancreatic Islet HMGB1 Release in Mice

**Figure 4:**
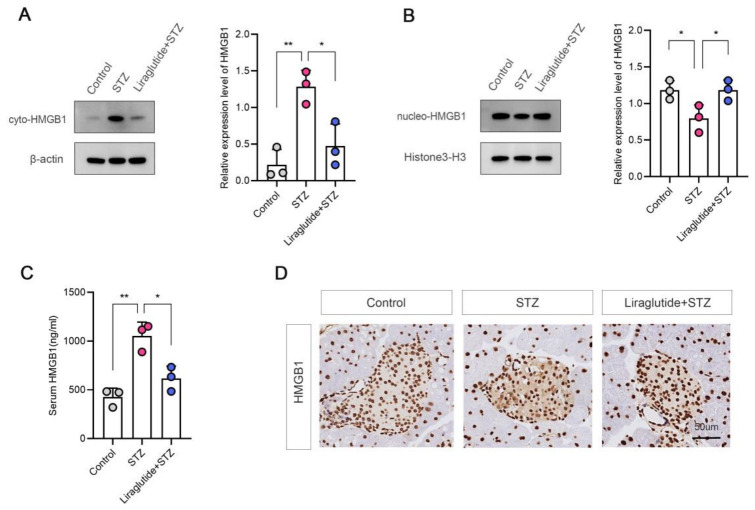
Liraglutide inhibited STZ-induced HMGB1 release from the mouse pancreas. (**A**) Western blot detection of HMGB1 expression in the cytoplasm of pancreatic tissues, by group (*n* = 3). (**B**) Western blot detection of HMGB1 expression in the nucleus of pancreatic tissues, by group (*n* = 3). (**C**) ELISA detection of serum HMGB1 expression in mice (*n* = 3). (**D**) Immunohistochemical detection of HMGB1 expression and distribution in mouse pancreas tissues (bar = 50 μm). * *p* < 0.05, ** *p* < 0.01.

HMGB1 is strongly correlated with pancreatic islet injury. When β-cells undergo stress or damage, HMGB1 is actively or passively released from the nucleus to the cytoplasm and blood circulation, inducing a series of inflammatory responses. To further investigate the protective mechanism of liraglutide on pancreatic islet cells, we examined the expression and distribution of HMGB1. Our results revealed that cytoplasmic HMGB1 was significantly increased in the STZ group, as compared with the control group. In the Liraglutide + STZ group, cytoplasmic HMGB1 was significantly reduced as compared with the STZ group (Figure 4A). Compared with the other two groups, intranuclear HMGB1 was reduced (Figure 4B), and serum HMGB1 was significantly increased (Figure 4C) in the STZ group. Immunohistochemistry revealed that HMGB1 was mainly located in the nucleus of pancreatic cells, with almost no HMGB1 found in the cytoplasm of the control and Liraglutide + STZ groups. Conversely, in addition to being located in the cell nucleus, HMGB1 was significantly increased in the cytoplasm of the STZ group (Figure 4D). This suggests that liraglutide can inhibit STZ-induced HMGB1 release, and HMGB1 may play an important role in STZ-induced pancreatic islet injury.

### 3.4. Liraglutide Inhibits STZ-Induced HMGB1 Release from NIT-1 Cells

**Figure 5:**
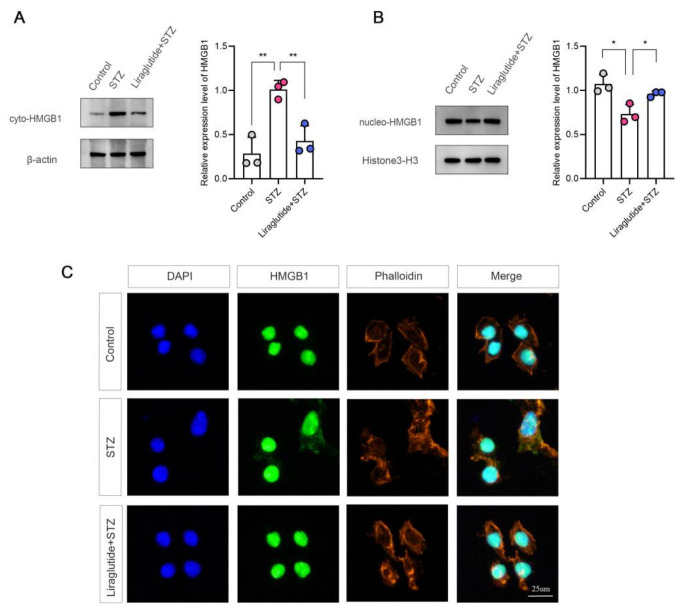
Liraglutide inhibits STZ-induced HMGB1 release from NIT-1 cells. (**A**) Western blot detection of HMGB1 expression in the cytoplasm of NIT-1 cells (*n* = 3). (**B**) Western blot detection of HMGB1 expression in the nucleus of NIT-1 cells (*n* = 3). (**C**) Immunofluorescence detection of HMGB1 expression and distribution in NIT-1 cells (bar = 25 μm). * *p* < 0.05, ** *p* < 0.01.

To further verify whether liraglutide inhibits STZ-induced HMGB1 release, the effects of liraglutide and STZ on mouse islet β-cell line NIT-1 were investigated. The results showed that compared with the control and the Liraglutide + STZ groups, HMGB1 protein content increased in the cytoplasm (Figure 5A) and decreased in the nucleus (Figure 5B) of NIT-1 cells in the STZ group. Immunofluorescence revealed that HMGB1 was localized to the nucleus of NIT-1 cells in the control and Liraglutide + STZ groups. In contrast, HMGB1 was released from the nucleus into the cytoplasm in the STZ group (Figure 5C). Meanwhile, compared with the control group, the intracellular cAMP level and p-CREB protein expression level in the STZ-treated group were significantly reduced. In contrast, compared with the STZ group, both cAMP level and p-CREB protein expression were significantly elevated in the liraglutide + STZ group (Supplementary Figure S1A–C). These findings indicate that liraglutide reverses STZ-induced inhibition of the cAMP/PKA/CREB signaling pathway. Collectively, these results suggest that liraglutide may inhibit HMGB1 translocation through activation of the GLP-1R/cAMP/PKA/CREB signaling pathway.

### 3.5. Liraglutide Alleviates STZ-Induced Islet Injury by Inhibiting HMGB1 Release

**Figure 6:**
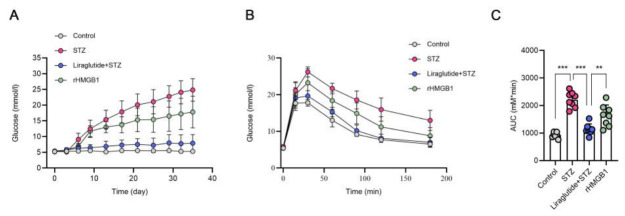
Recombinant HMGB1 reduces the protective effect of liraglutide. (**A**) Fasting blood glucose levels after treatment, by group (*n* = 10). (**B**) Glucose tolerance after treatment by group (*n* = 8). (**C**) Comparison of area under the curve, by group (*n* = 8). ** *p* < 0.01, *** *p* < 0.001.

We used recombinant HMGB1 (rHMGB1) protein to further investigate whether liraglutide protects pancreatic islet cells by inhibiting HMGB1 release. Specifically, after STZ was injected into the mice, an additional 20 μg of rHMGB1 per mouse was injected through the tail vein to observe whether the original protective effect of liraglutide on the pancreas was reduced with the additional increase of HMGB1. We found that mice in the rHMGB1 group had significantly higher fasting blood glucose levels (Figure 6A) and increased impaired glucose tolerance (Figure 6B,C) as compared with the Liraglutide + STZ group. This suggests that recombinant HMGB1 attenuates liraglutide’s islet-protective effects. Furthermore, compared with the control group, STZ treatment resulted in decreased cell viability, increased HMGB1 levels in the cell culture supernatant, and markedly elevated expression of the apoptotic marker Bax. In contrast, the Liraglutide + STZ, Glycyrrhizin + STZ, and Liraglutide + Glycyrrhizin + STZ groups all significantly ameliorated apoptosis, with no statistically significant differences in protective effects among these three groups (Supplementary Figure S2A–F). Collectively, these findings demonstrate that liraglutide alleviates STZ-induced islet injury by inhibiting HMGB1 release, and that this protective effect is primarily dependent on the HMGB1 signaling pathway.

### 3.6. Liraglutide Affects Pancreatic TLR4 Expression and Reduces Inflammation In Vivo

**Figure 7:**
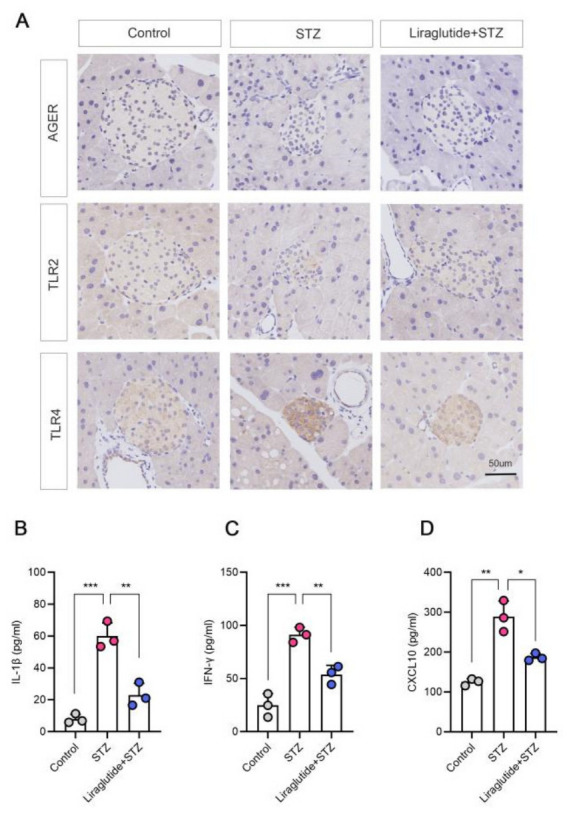
Liraglutide reduces STZ-induced TLR4 expression in mouse pancreas and inflammatory factor levels in vivo. (**A**) Immunohistochemistry detection of the expression of HMGB1 receptor AGER, TLR4, and TLR2 in pancreatic tissues. (**B**) ELISA detection of IL-1β expression, by group (*n* = 3). (**C**) ELISA detection of IFN-γ expression, by group (*n* = 3). (**D**) ELISA detection of CXCL10 expression, by group (*n* = 3). * *p* < 0.05, ** *p* < 0.01, *** *p* < 0.001.

To investigate whether liraglutide inhibits HMGB1 receptors, this study further examined the expression of HMGB1 receptors (AGER, TLR4, TLR2) by immunohistochemistry in the pancreatic tissues of the treatment and control groups (Figure 7A). There were no significant differences in AGER and TLR2 expression among the three groups; however, TLR4 expression was significantly higher in the STZ group (Supplementary Figure S3A–C). Western blot analysis revealed that the protein expression level of TLR4 in the pancreatic tissue of mice in the STZ group was significantly upregulated compared to the control group. In contrast, the Liraglutide + STZ group exhibited a marked decrease in TLR4 protein expression relative to the STZ group alone (Supplementary Figure S3D–G). The western blot results also revealed that p-P65 protein expression was significantly increased following STZ treatment compared with the control group, whereas liraglutide intervention reduced its expression level (Supplementary Figure S3H). Evaluation of in vivo inflammation levels in mice revealed that the serum levels of pro-inflammatory cytokines IFN-γ and IL-1β and inflammatory chemokine CXCL10 were significantly higher in the STZ group than in the control group. Moreover, inflammatory factor levels were all significantly reduced in the Liraglutide + STZ group as compared with the STZ group (Figure 7B–D). This suggests that liraglutide may have an anti-inflammatory effect.

### 3.7. Avexitide Partially Eliminates Liraglutide’s Islet-Protective Effects

**Figure 8:**
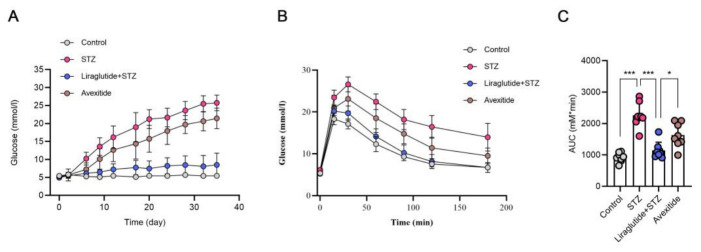
Avexitide partially eliminates the protective effect of liraglutide. (**A**) Fasting blood glucose levels following treatment, by group (*n* = 10). (**B**) Glucose tolerance levels following treatment, by group (*n* = 8). (**C**) Comparison of area under the curve (*n* = 8). * *p* < 0.05, *** *p* < 0.001.

To investigate whether liraglutide exerts its protective effect via GLP-1 receptors, mice were additionally administered 250 μg/kg of Avexitide, a GLP-1 receptor antagonist, 30 min before liraglutide injection. Compared with the Liraglutide + STZ group, fasting blood glucose levels were significantly elevated in the Avexitide group after antagonism of GLP-1R (Figure 8A). The degree of impaired glucose tolerance in the Avexitide group was increased (Figure 8B), and the area under the curve was between the STZ group and the Liraglutide + STZ group (Figure 8C). This indicates that the protective effect of liraglutide may be partially eliminated after blocking GLP-1 receptors with Avexitide, suggesting that the GLP-1 receptor could mediate liraglutide’s islet-protective effect.

## 4. Discussion

As a GLP-1 receptor agonist, liraglutide can improve pancreatic β-cell function, promote the proliferation and inhibit the death of pancreatic β-cells, and blood glucose homeostasis. Liraglutide has been approved and marketed for the treatment of type 2 diabetes mellitus. However, the protective effect of liraglutide in acute islet injury and its mechanism remain unclear. In the present study, we demonstrated for the first time that liraglutide could potently protect against STZ-induced islet injury, suggesting that liraglutide has potential for the treatment of acute pancreatic injury diseases.

In recent years, clinical trials have reported that GLP-1 receptor agonists have an adjunctive therapeutic effect in the treatment of type 1 diabetes [27]. Compared with insulin alone, the combination of insulin and GLP-1 agonists can better control fasting blood glucose and glycated hemoglobin levels [28,29,30]. Incretin-based therapy has a better effect on HbA1c reduction in patients with type 1 diabetes [31]. Basic studies have reported that GLP-1 receptor agonists can reduce the level of pancreatic islet inflammation, improve glucose tolerance, and delay the onset of diabetes in NOD mice [32,33]. This study found that liraglutide has a strong protective effect on STZ-induced islet damage, can significantly improve the islet function, and markedly reduce the blood glucose levels and the incidence of type 1 diabetes mellitus in mice. Notably, short-term pretreatment reduced the incidence of diabetes to 15.4%. Continuous administration may produce stronger protective effects, and future studies could further evaluate the benefits of long-term liraglutide treatment. Taken together, these findings suggest that liraglutide holds a strong promise in protecting against islet damage and preventing the progression of type 1 diabetes.

Compared to healthy patients, higher levels of HMGB1 have been detected in the serum of patients with diabetes [34]. Exposure to high levels of glucose resulted in increased HMGB1 expression or release [35,36], while neutralization of HMGB1 alleviated the progression of autoimmune diabetes in NOD mice [37]. These studies suggest that HMGB1 is closely related to islet damage and diabetes progression. As another GLP-1 receptor agonist, Exendin-4 has been demonstrated to ameliorate diabetes and associated islet β-cell damage [38,39]. Notably, Exendin-4 attenuates high glucose-induced cardiomyocyte injury and inflammation by suppressing HMGB1 [18]. Given the pivotal role of HMGB1 in islet injury, we speculate that Exendin-4 may exert its glucose-lowering and islet-protective effects through a similar mechanism—inhibition of HMGB1 and subsequent inflammatory cascades. Nevertheless, whether liraglutide’s protection against STZ-induced islet damage is mediated through HMGB1 suppression requires further investigation. In the present study, STZ triggered HMGB1 nucleocytoplasmic shuttling and induced inflammatory responses in mice, while liraglutide significantly inhibited this effect. In contrast, the protective effect of liraglutide on pancreatic islets was significantly reduced by the use of recombinant HMGB1 protein. This suggests that the nuclear-cytoplasmic translocation and release of HMGB1 play an important role in islet damage, and targeted intervention of HMGB1 may be a novel strategy to protect pancreatic islet cells.

Under stress conditions, HMGB1 is released from the nucleus and acts as an extracellular signal conferred by the HMGB1 receptors [40]. In this study, we detected the expression of HMGB1 receptors, and found that TLR4 expression was elevated in the STZ group, and liraglutide pretreatment could inhibit the increased expression of TLR4. TLR4 is a pattern recognition receptor, and targeted blockade of the TLR4 signaling pathway reduces diabetes-associated inflammatory responses [41], whereas activation of TLR4 signaling induces pro-inflammatory cytokine production that exacerbates β-cell damage [42]. Thus, the protective effect of liraglutide on acute islet injury may be related to the inhibition of the HMGB1-TLR4 signaling pathway. In addition, we found that the protective effect of liraglutide was partially eliminated after blocking GLP-1 receptor with Avexitide, suggesting that GLP-1 receptor may mediate this protective effect. How the intracellular signal transmitted by GLP-1/GLP-1 receptor exerts its inhibitory effect on the migration and release of nuclear HMGB1 still needs further study.

Inflammatory responses play an essential role in the pathogenesis of diabetes [43]. The release of HMGB1 induces a series of inflammatory responses [44]. Increased levels of IL-1β are commonly detected in patients with type 1 diabetes [45], and damaged pancreatic β-cells secrete large amounts of the chemokine CXCL10 [46]. As a key molecule that coordinates the entry of lymphocytes into the site of inflammation, CXCL10 recruits self-effector T cells to attack pancreatic islet tissues, further exacerbating the damage [47]. Moreover, IFN-γ is an important molecule that stimulates CXCL10 production by β-cells and induces an inflammatory response [48]. This study also found that the serum levels of IL-1β, IFN-γ, and CXCL10 were all elevated in STZ-treated control mice. However, liraglutide pretreatment significantly inhibited the increase of these islet injury-associated inflammatory factors induced by STZ, and this inhibitory effect may be related to the inhibition of HMGB1 release. Importantly, in addition to its pro-inflammatory activity, HMGB1 may also participate in tissue repair processes. HMGB1 promotes tissue regeneration by chemoattracting and activating stem cells, as well as stimulating the proliferation and differentiation of tissue-associated resident stem cells, such as fibroblasts, endothelial cells, and vascular smooth muscle cells [49,50,51]. Studies have confirmed that HMGB1 is a pro-angiogenic factor, which likely promotes angiogenesis by regulating blood vessel growth through the TLR4 and RAGE receptors [52,53]. Future investigations should explore the role of HMGB1 in islet repair to comprehensively elucidate the bidirectional regulatory mechanisms of this molecule.

Several limitations of this study should be acknowledged. First, while we demonstrate that liraglutide inhibits STZ-induced HMGB1 release and that exogenous HMGB1 diminishes liraglutide’s protective effects, there is no direct loss-of-function of HMGB1 downstream signaling. Second, it should be noted that downstream signaling of GLP-1R was assessed only in cultured cell lines, and no measurements were performed in primary mouse islets. Future studies should address these limitations by including TLR4 knockdown/inhibition experiments, HMGB1 acetylation analysis and direct measurements of GLP-1R signaling in isolated islets in order to more comprehensively elucidate the role and regulatory mechanism of HMGB1 in the protective effect of liraglutide against islet injury.

## 5. Conclusions

In conclusion, this study demonstrated for the first time that liraglutide has a strong protective effect on STZ-induced acute islet injury, most likely through the inhibition of HMGB1 release from pancreatic islet cells, which provide a theoretical basis for the use of liraglutide as a pancreatic islet damage protector.

## Data Availability

The original contributions presented in this study are included in the article/Supplementary Materials. Further inquiries can be directed to the corresponding authors.

## Supplementary Materials

The following supporting information can be downloaded at https://www.mdpi.com/article/10.3390/cells15131203/s1, Figure S1: Liraglutide reverses STZ-induced inhibition of the cAMP/PKA/CREB signaling pathway; Figure S2: Liraglutide alleviates STZ-induced islet injury by inhibiting HMGB1 release; Figure S3: Liraglutide downregulates STZ-induced TLR4 expression at the protein level; Figure S4: ELISA detection of HMGB1 levels in serum at 12 h, 24 h, and 48 h after injection of recombinant HMGB1 protein; Table S1: Primer sequences.
