# Supplementary material for: Liraglutide Potently Protects Against Streptozotocin-Induced Islet Injury Associated with Inhibition of HMGB1 Release
Source: Cells. 2026 Jul 2;15(13):1203. doi: 10.3390/cells15131203 (PMC13359402; doi:10.3390/cells15131203)
Supplement: Supplementary file 1 [file cells-15-01203-s001.zip › cells-4303218-supplementary.pdf]

Supplementary Figure and Table

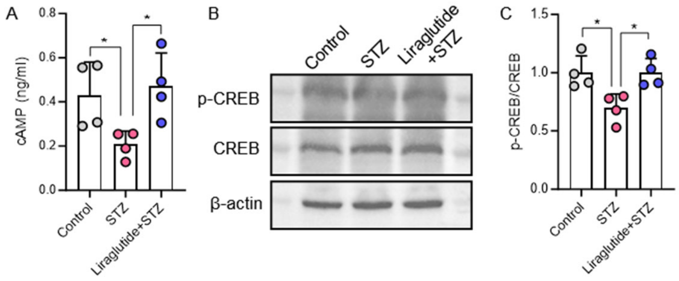


Supplementary Figure S1. Liraglutide reverses STZ-induced inhibition of the cAMP/PKA/CREB signaling pathway. (A) Detection of cAMP levels in NIT-1 Cells across groups (n=4). (B-C) Western blot detection of the protein expression levels of p-CREB and CREB in NIT-1 Cells from each group (n=4).*P<0.05.

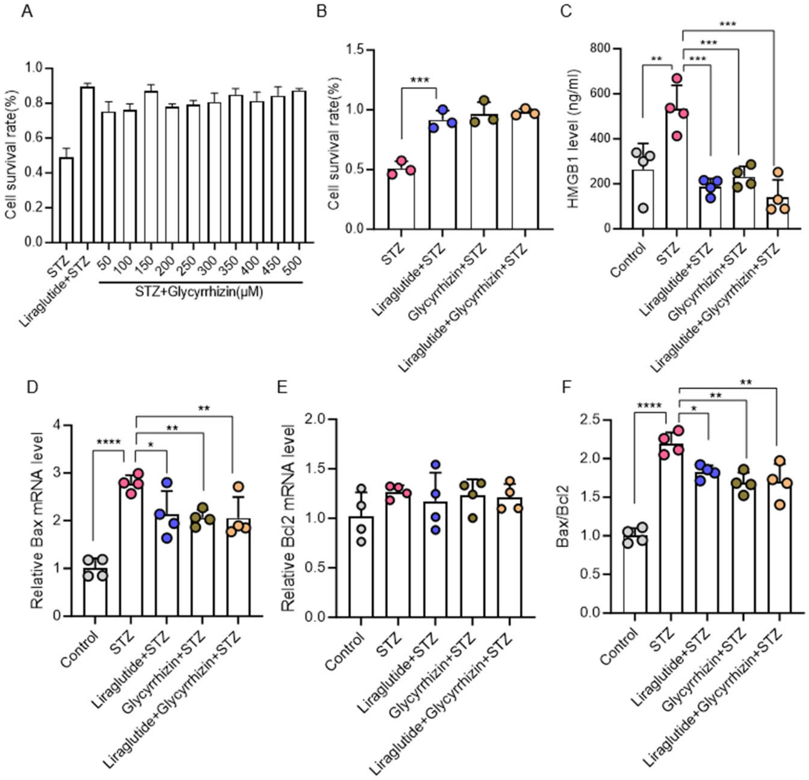


Supplementary Figure S2. Liraglutide alleviates STZ-induced islet injury by inhibiting HMGB1 release. (A) CCK-8 assay detection of cell viability of NIT-1 cells co-treated with various concentrations of

glycyrrhizin and STZ (n=3). (B) CCK-8 assay detection of cell viability of each treatment group (n=3). (C) ELISA detection of HMGB1 levels in the cell culture supernatant of each treatment group (n=4). (D-F) QPCR detection of mRNA expression levels of Bax and Bcl-2 in each treatment group (n=4).*P<0.05, **P<0.01,***P<0.001.

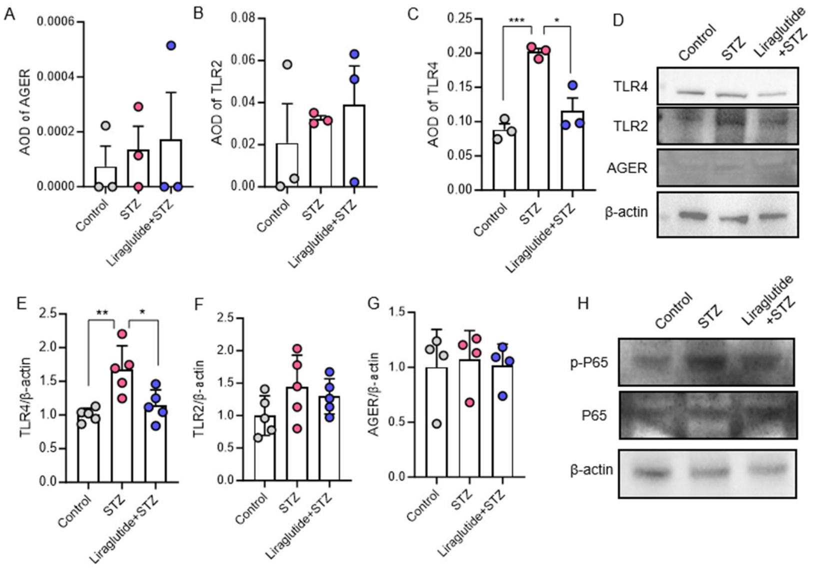


Supplementary Figure S3. Liraglutide downregulates STZ-induced TLR4 expression at the protein level. (A-C) The quantitative analysis of AGER, TLR2 and TLR4 immunohistochemistry staining (n=3). (D-G) Western blot detection and the quantitative analysis of the protein expression levels of AGER,TLR2 and TLR4 in pancreatic tissues across groups (n=4-5). (H) Western blot detection of the protein expression levels of p-P65 and P65 in pancreatic tissues across groups (n=4). *P<0.05, **P<0.01,***P<0.001.

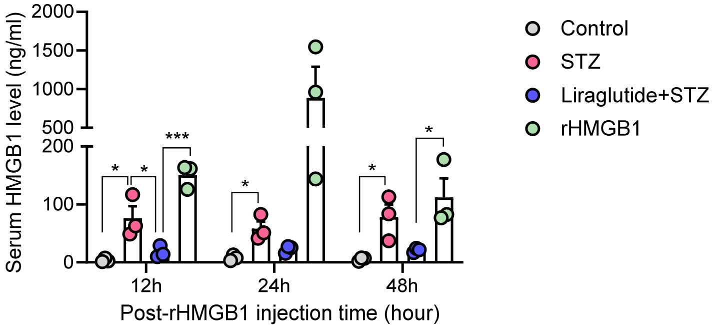


Supplementary Figure S4. ELISA detection of HMGB1 levels in serum at 12h, 24h, and 48h after injection of recombinant HMGB1 protein (n=3). *P<0.05, ***P<0.001.

Supplementary Table S1. Primer sequences

| Gene | Forward | Reverse |
|---|---|---|
| *Bax* | 5′-CGTGGTTGCCCTCTTCTACTTT-3′ | 5′-GATCAGCTCGGGCACTTTAGTG-3′ |
| *Bcl2* | 5′-GTCATCCACAGAGAGCGATGTT-3′ | 5′-GTTCATCTCGGAGCCTGTAGTG-3′ |
| *Beta-actin* | 5′-ACTGTCGAGTCGCGTCC-3′ | 5′-GATGACTTCTCTCGTCGCTA-3′ |
